# Genome‐Wide Analysis of circular RNAs and validation of hsa_circ_0006719 as a potential novel diagnostic biomarker in congenital scoliosis patients

**DOI:** 10.1111/jcmm.15370

**Published:** 2020-05-12

**Authors:** Gang Liu, Jianxiong Shen, Chong Chen, Yang Jiao, Zheng Li, Haining Tan, Youxi Lin, Tianhua Rong

**Affiliations:** ^1^ Department of Orthopedic Surgery Peking Union Medical College Hospital Peking Union Medical College and Chinese Academy of Medical Sciences Beijing China; ^2^ Beijing Key Laboratory for Genetic Research of Skeletal Deformity Beijing China; ^3^ Medical Research Center of Orthopedics Chinese Academy of Medical Sciences Beijing China; ^4^ Department of Spine Surgery Orthopedics Center of Guangdong Provincial People's Hospital and Guangdong Academy of Medical Sciences Guangzhou China

**Keywords:** biomarker, circular RNA, congenital scoliosis, diagnosis, genome‐wide circRNA sequencing

## Abstract

Congenital scoliosis (CS) is a form of spinal curvature resulting from anomalous development of vertebrae. Recent studies demonstrated that circRNAs could serve as potential biomarkers of disease diagnosis. Genome‐wide circRNAs expression in seven CS patients and three healthy controls was initially detected. Bioinformatics analysis was conducted to explore the potential pathological pathway of CS. Quantitative PCR (qPCR) was performed to validate the selected circRNAs in the replication cohort with 32 CS patients and 30 healthy controls. Logistic regression controlling for gender was conducted to compare the expression difference. Receiver operating characteristic (ROC) curve analysis was performed to evaluate the diagnostic value. Twenty‐two differentially expressed circRNAs were filtered from genome‐wide circRNA sequencing. Seven circRNAs were validated by qPCR. Only hsa_circ_0006719 was confirmed to have a higher expression level in the CS group than the healthy control group (*P* = 0.036). Receiver operating characteristic curve also suggested that hsa_circ_0006719 had significant diagnostic value for CS (AUC = 0.739, *P* = 0.001). We described the first study of circRNAs in CS and validated hsa_circ_0006719 as a potential novel diagnostic biomarker of CS.

## 1 BACKGROUND

Congenital scoliosis (CS) is a form of spinal malformation resulting from abnormal axial bone development in embryo. The incidence of CS is approximately 0.5‐1 per 1000 newborn infants.^1^, ^2^, ^3^ CS patients always have multiple system deformity, such as missing ribs,^4^ split cord malformation^5^ and pulmonary dysfunction,^6^ which physically and psychologically affect their lives and daily activities. Although the studies to explore the pathogenesis of CS have been conducted for many years, the aetiology is still elusive. Previous studies have proved that genetic factors could lead to the development of CS, such as rearrangement of chromosome 16p11.2,^7^ mutation of *TBX6* ^8^ or *FBN1*.^9^ There is also evidence that environmental factors could contribute to the development of CS. Li et al^10^ found that vitamin A deficiency in pregnancy may induce CS in rats. Some researchers illustrated that hypoxia and high altitude are associated with a higher risk of CS.^1^, ^11^ However, there were few types of research focusing on the linkage of these aetiological factors. Thus, it is urgent and necessary to identify novel molecular markers of the mechanism, even for diagnosis and therapeutics.

Circular RNAs (circRNAs) are a class of non‐coding RNAs that manifested with stable circular RNA structure.^12^ Recent studies have reported that circRNAs play a pivotal role in different kinds of diseases. CircRNAs could act as efficient microRNA sponges,^13^ which function as post‐transcriptional regulators.^14^ Some researchers also found that some circRNAs could work as protein sponges,^15^ which can influence gene regulation. Other studies also found in cellular responses to environmental stress, some circRNAs can be translated.^16^, ^17^ Because of the unusually stable circular structure, circRNAs were also reported to be promising diagnostic biomarkers in different diseases.^18^, ^19^ There are very few studies focusing on circRNAs and CS. Our previous study has identified significantly different expressed circRNAs in vitamin A deficiency‐induced CS rats.^20^ However, the function and characteristics of circRNAs in CS patients are still unclear.

In this study, we enrolled seven patients and three healthy controls to identify and compare the expression of genome‐wide circRNAs. After filtering, annotation and validation, we compared the differentially expressed circRNAs aiming to find the potential diagnostic and therapeutic biomarkers of CS.

## 2 MATERIALS AND METHODS

### 2.1 Patients and materials

Seven patients diagnosed as CS and three healthy controls were recruited from Peking Union Medical College Hospital (PUMCH). Thirty‐two CS patients and 30 healthy controls were enrolled as a replication cohort. The inclusion and exclusion criteria of CS were as follows.

#### 2.1.1 Inclusion criteria

1. Patients diagnosed as CS.
2. Age of onset under 18 years old.
3. Having complete imaging data, including X‐ray, three‐dimensional imaging of the spine CT or spinal MRI.

#### 2.1.2 Exclusion criteria

1. Other types of scoliosis including adolescent idiopathic scoliosis, neuromuscular scoliosis, scoliosis secondary to skeletal dysplasia or connective tissue abnormalities.
2. Incomplete imaging data.
3. Having a chronic disease that influenced skeletal development.

All patients were diagnosed as CS by at least two experienced orthopaedic surgeons. The clinical information of the study subjects was summarized in Table 1. Written informed consent was obtained from all the participants or their guardians. All controls were healthy without any spinal deformity or other diseases. The Ethical Review Board of Peking Union Medical College Hospital approved this study (Protocol Number: JS‐1901).

**TABLE 1:** Basic characteristics of participants in the study

Characteristics	Discovery cohort		Replication cohort	
	CS group (n = 7)	Control group (n = 3)	CS group (n = 32)	Control group (n = 30)
Mean age (y)	13.57	26.67	14.49	23.67
Gender M:F	1:6	3:0	17:15	10:20
Main Cobb angle (°)	88.86	NA	56.26	NA

### 2.2 RNA extraction and Genome‐wide circRNAs sequencing

Total RNA was extracted from the peripheral blood using a Qiagen PAXgene Blood miRNA Kit (QIAGEN, Eastwin Scientific, Inc, Beijing, China) according to the manufacturer's instructions. The quality and integrity of the extracted RNA were evaluated using an RNA 6000 Nano Lab Chip Kit (Agilent Technologies, CA, USA) and an Agilent 2100 Bioanalyzer (Agilent Technologies, CA, USA). The ribosomal RNAs were removed using a Ribo‐Zero Gold rRNA Removal Kit (Human/Mouse/Rat) (Epicenter Company, Madison, WI, USA). The linear RNAs were digested by RNase R to separate the circRNAs. The RNAs were then reverse‐transcribed to create the final cDNA library using the mRNA‐Seq sample preparations kit (Illumina, San Diego, CA, USA). Sequencing was carried out by HiSeq X‐Ten with 150‐bp pair‐end reads mode.

### 2.3 Bioinformatics analysis and annotation of circRNAs

The low‐quality reads were removed, and the clean reads were aligned to the reference human genome (hg19). Based on the tool of find_circ,^14^ find_circ_enhance was used to identify circRNAs with default parameters (circRNA read count ≥2 and unique alignment reads ≥2). Filtered circRNAs were used for further annotation. After comparing to circBase, novel circRNA and known circRNA were separated. Transcripts per million clean tags (TPM) were applied to quantify the expression of circRNAs.^21^ Differentially expressed circRNAs were estimated by DESeq2.^22^ The expression level was considered significantly different when |log2 fold change| > 1 and *P* value < 0.05. Functional enrichment analysis of the differentially expressed circRNA‐related genes at Gene Ontology (GO) and Kyoto Encyclopedia of Genes and Genomes (KEGG) was performed using clusterProfiler (v3.6.0) Bioconductor package.

### 2.4 Validation of selected circRNAs

Quantitative PCR (qPCR) was performed to validate the circRNAs identified by the bioinformatics analysis. Divergent primers were designed using circPimer (v1.2). First‐strand cDNA was synthesized using SuperScript III First‐Strand (Thermo Fisher Scientific, Beijing, China) according to the manufacturer's instructions. qPCR condition was as follows: an initial 1‐min denaturation at 95°C (Ramp Rate 4.4°C/s) followed by 40 cycles of 15 seconds at 95°C, 15 seconds at the appropriate annealing temperature (depending on the divergent primer set used) and 30 seconds extension at 72°C, then 5 seconds at 95°C, 60 seconds at 60 and 95°C (Ramp rate 0.11°C/s) for melt curve analysis, with a final cooling at 50°C for 30 seconds. For candidate circRNAs, the PCR products were separated from the electrophoresis agarose gel. Sanger sequencing was conducted to validate the back‐spliced site of each circRNAs. Each qPCR of each circRNA was replicated three times using β‐actin as the internal control for normalization of expression. The ΔΔCt method was performed to calculate the relative expression of circRNAs.

### 2.5 Statistical analysis

Data of qPCR were analysed using SPSS software (16.0 version, SPSS Inc, Chicago, IL, USA). The expression differences of qPCR were evaluated using logistic regression controlling for gender. Receiver operating characteristic (ROC) curve was performed to estimate the diagnostic application of circRNA.

## 3 RESULTS

### 3.1 Genome‐wide circRNA expression profiles

**FIGURE 1:**
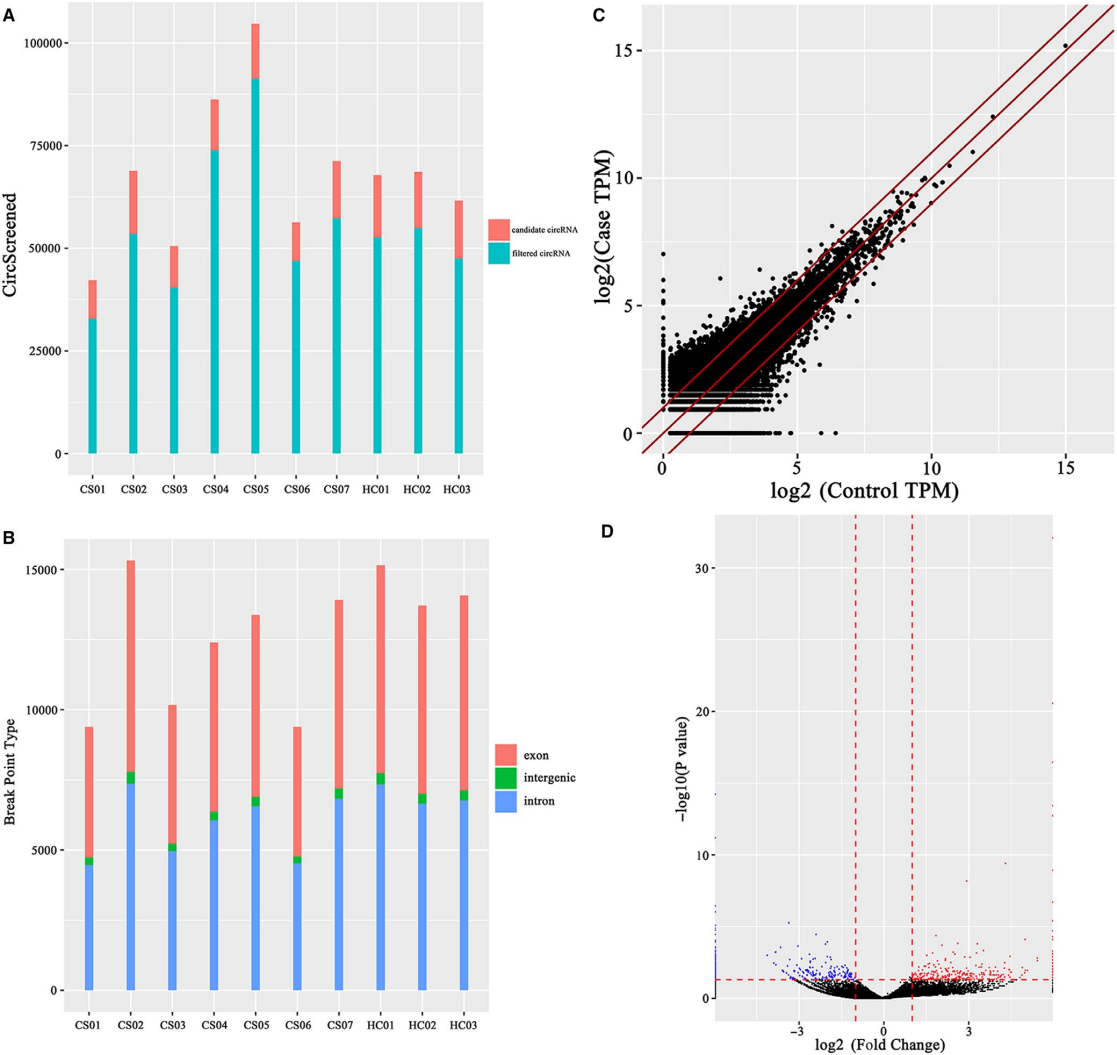
Characteristics of genome‐wide circRNA expression profiles of the CS group and healthy control group. A, The total number of filtered and candidate circRNAs screened. B, The source of circRNAs based on the breakpoint. C, The scatterplot of the differentially expressed circRNAs. The circRNAs above the top red line and below the bottom red line indicate more than a 1.0‐fold change between the two groups. D, The volcano plot of circRNA expression profile. The vertical lines correspond to 1.0‐fold change up‐regulation and down‐regulation and the horizontal line represents *P* = 0.05

There were 551,278 circRNAs identified by circRNA sequencing in seven CS patients and three healthy controls. After filtering, there were 126 907 candidate circRNAs left for further analysis (Figure 1A). In order to identify the source of those circRNAs, breakpoint type was also annotated (Figure 1B). Most of them were derived from exonic or intronic circRNAs. Differentially expressed circRNAs were estimated by the fold changes (FC) of circRNA expression (Figure 1C). In total, 394 circRNAs with significantly different expression level were analysed by the volcano plot (Figure 1D, Table S1).

**FIGURE 2:**
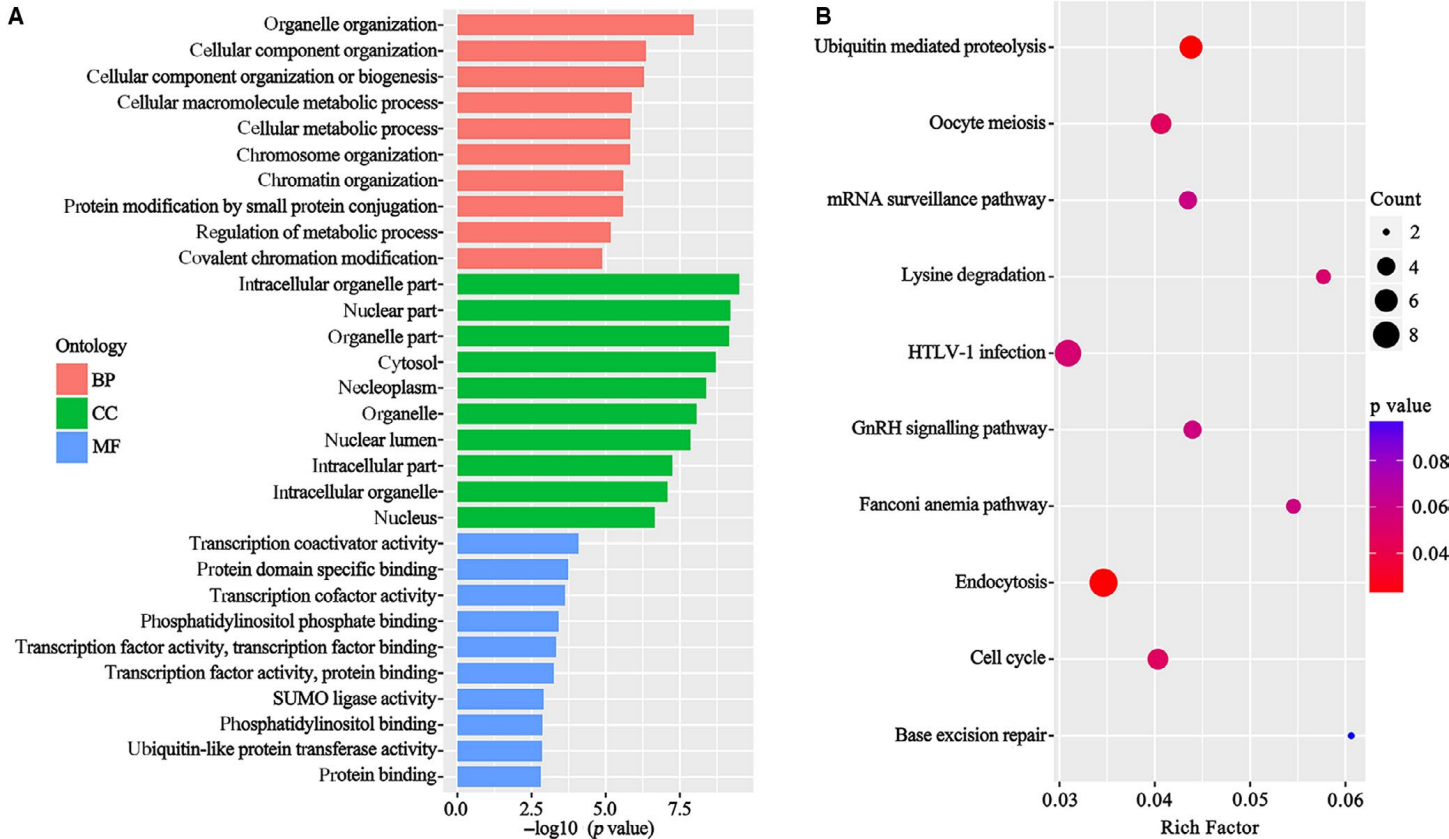
GO and KEGG analysis of the differentially expressed circRNA‐related genes in the CS group and healthy control group. A, The enriched results of biological process (BP), cellular component (CC) and molecular function (MF) by GO analysis. B, Pathways enriched in the KEGG analysis

We enriched the differentially expressed circRNA‐related genes and then subjected these genes to GO annotation and KEGG pathway analysis. It indicated that the circRNA‐related genes were enriched in several pathways. The top outcomes of GO enrichment were organelle organization in biological process (BP), intracellular organelle part in cellular component (CC) and transcription coactivator activity in molecular function (MF) (Figure 2A). In the KEGG pathway analysis, three pathways with the most significant association were the ubiquitin‐mediated proteolysis signalling pathway, endocytosis signalling pathway and oocyte meiosis signalling pathway (Figure 2B), indicating that these pathways could be associated with CS.

### 3.2 Validation of selected circRNAs

**FIGURE 3:**
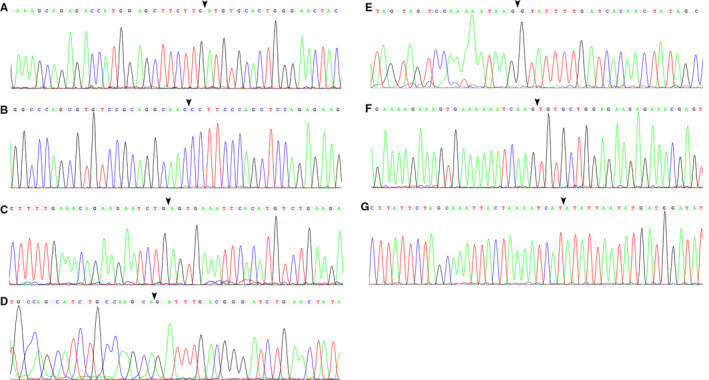
Sanger sequencing of hsa_circ_0006856 (A), hsa_circ_0006719 (B), hsa_circ_0006208 (C), hsa_circ_0002785 (D), hsa_circ_0002692 (E), hsa_circ_0002372 (F) and hsa_circ_0000225 (G). The arrows represented the back‐spliced sites

Combing with the *P* value and the read count of each circRNAs, we selected 22 circRNAs with the most stable and significant different expression for validation (Table 2). Divergent primers were designed (Table S2), and qPCR was performed, and the back‐spliced sites of seven circRNAs were confirmed by Sanger sequencing including hsa_circ_0006856, hsa_circ_0006719, hsa_circ_0006208, hsa_circ_0002785, hsa_circ_0002692, hsa_circ_0002372 and hsa_circ_0000225 (Figure 3). Then, we compared the expression level with ΔΔCt method (Table S3). All of those seven circRNAs had higher expression levels in the CS group than the healthy control (HC) group (Table S4). After compared with the expression level of the discovery cohort, only hsa_circ_0006719 had the same expression tendency in the replication cohort. After logistic regression controlling for gender, we found that the expression level was statistically significant different between the CS group and the HC group (95% CI: 0.59‐0.98, *P* = 0.036; Table 3).

**TABLE 2:** The 22 selected circRNAs with the most stable and significant different expression

CircRNA	Control	Case	log2(fold change)	*P*
hsa_circ_0006719	77.29	276.76	1.84	4.25E−05
hsa_circ_0037173	2.17	11.78	2.44	0.01
chr3_105389076_105404310_‐	1.01	10.41	3.37	0.01
hsa_circ_0001243	1.24	9.48	2.94	0.02
hsa_circ_0041267	1.22	8.58	2.82	0.02
chr19_50840790_50865349_‐	10.55	30.27	1.52	0.02
hsa_circ_0006208	55.92	5.43	−3.36	5.41E−06
hsa_circ_0002692	120.66	22.85	−2.4	3.54E−05
hsa_circ_0000225	98.56	35.15	−1.49	0.01
chr5_72311452_72333042_+	15.36	2.69	−2.51	0.01
hsa_circ_0023233	12.17	1.34	−3.18	0.01
chr14_59730158_59758024_+	18.61	3.58	−2.38	0.01
hsa_circ_0002785	15.16	2.24	−2.76	0.01
hsa_circ_0007128	14.49	1.83	−2.99	0.01
chr9_3630937_3651867_+	27.01	5.89	−2.2	0.01
hsa_circ_0002372	13.75	2.25	−2.61	0.02
hsa_circ_0006856	69.08	27.04	−1.35	0.02
chr11_65202523_65211534_+	32.54	9.9	−1.72	0.02
chr9_3647337_3651867_+	87.83	35.67	−1.3	0.02
hsa_circ_0002280	12.89	2.25	−2.52	0.03
chr21_40578033_40584633_‐	44.01	17.36	−1.34	0.03
chr6_37250657_37284982_+	12.18	2.27	−2.43	0.04

**TABLE 3:** Logistic regression predicting likelihood of CS based on gender and hsa_circ_0006719

	*B*	SE	Wald	*df*	*P*	Odds Ratio	95% CI for Odds Ratio	
							Lower	Upper
Gender	−0.95	0.59	2.603	1	0.107	0.39	0.12	1.23
has_circ_0006719	−0.28	0.12	4.40	1	0.036	0.76	0.59	0.98
Constant	1.04	0.45	5.37	1	0.020	2.83		

Note

Gender is for males compared to females.

### 3.3 ROC curve analysis of hsa_circ_0006719 in CS patients

**FIGURE 4:**
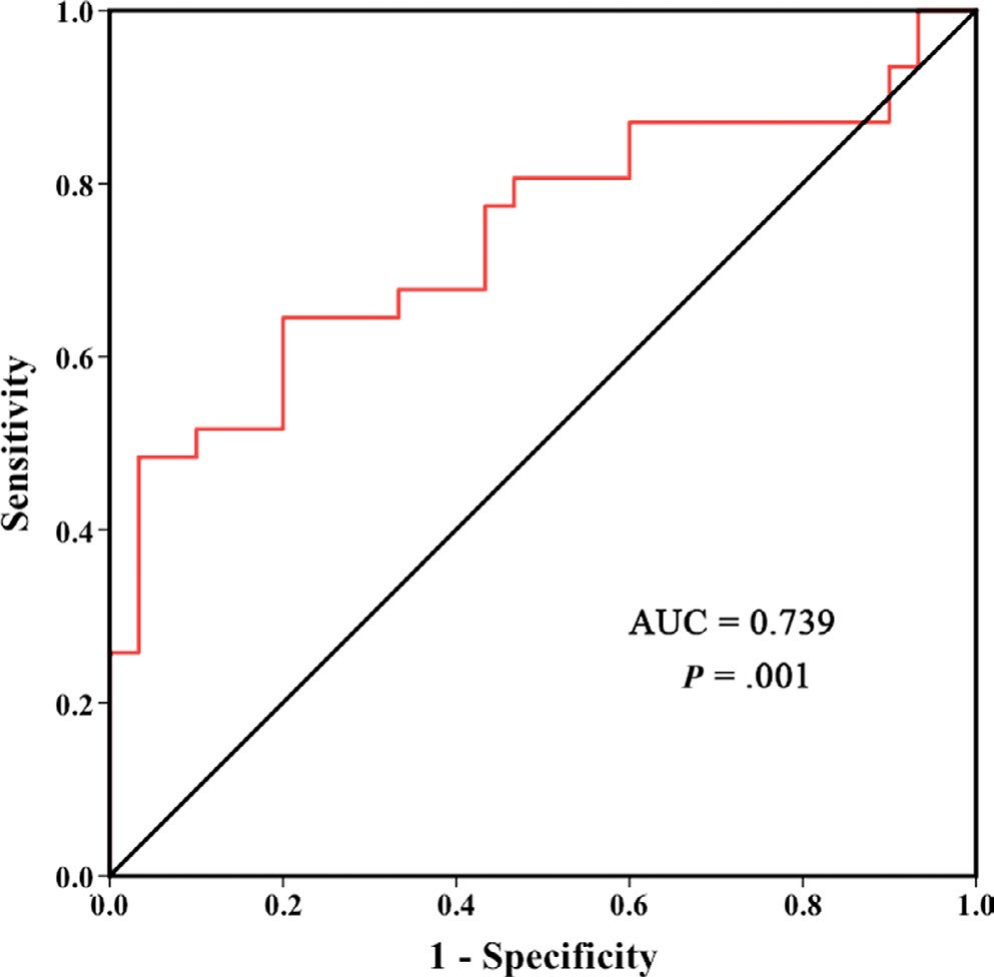
ROC curve analysis of hsa_circ_0006719 in CS patients. The AUC value is 0.739 with *P* value of 0.001

To estimate the diagnostic value of hsa_circ_0006719 as candidate biomarkers of CS, ROC curve analysis was performed. The area under the curve (AUC) was 0.739 (95% CI: 0.611‐0.866, *P* = 0.001) (Figure 4). Therefore, hsa_circ_0006719 may be a potential diagnostic biomarker for CS.

## 4 DISCUSSION

In this study, we performed genome‐wide circRNA sequencing and compared the circRNA expression profiles of peripheral blood from CS patients and healthy controls. Gene Ontology and KEGG pathway analysis were conducted to explore the potential function of the differentially expressed circRNAs. Previous studies have revealed that circRNAs are outputs of protein‐coding genes in eukaryotes.^23^, ^24^ They could function as post‐transcriptional regulators, including working as microRNA sponge and protein sponge, even some of them could translate into proteins. Thus, it is feasible to predict the functions of circRNAs via their related genes.^25^ It is postulated that GO and KEGG pathways are extensively used in predicting gene function and enrichment analysis.^26^, ^27^ According to GO annotation, we hypothesized that circRNAs might contribute to the development of CS by biological process (BP), intracellular organelle part in cellular component (CC) and transcription coactivator activity in molecular function (MF). Kyoto Encyclopedia of Genes and Genomes pathway analysis also indicated that differentially expressed circRNA‐related genes were enriched in the ubiquitin‐mediated proteolysis signalling pathway, endocytosis signalling pathway and other several pathways. Previous studies indicated that the ubiquitin‐mediated proteolysis signalling pathway is involved in osteogenic differentiation and may play essential roles in disorders manifested with scoliosis.^28^, ^29^ The endocytosis signalling pathway has also reported to play an pivotal role in the development of scoliosis.^30^, ^31^ We hypothesized that these differentially expressed circRNAs could regulate ubiquitin‐mediated proteolysis and endocytosis signalling pathway, which could influence the development of CS.

To explore specific circRNAs involved in CS, we compared the expression level in the replication cohort. We selected 22 circRNAs with the most stable and significant different expression for the validation. Seven circRNAs were confirmed, and only hsa_circ_0006719 had higher expression level in the CS group than the HC group with significant difference. Moreover, we conducted ROC curve analysis to evaluate the value of hsa_circ_0006719 for differentiating CS and HC. The results suggested that hsa_circ_0006719 was a potential novel diagnostic biomarker of CS. hsa_circ_0006719 originates from the exon 3 and intron 3 of gene *VKORC1* with the spliced sequence length of 568 bases. *VKORC1* locates in 16p11.2 encoding vitamin K epoxide reductase (VKOR) complex subunit 1. VKORC1 plays an important role in vitamin K metabolism. It is a catalytic subunit of the VKOR complex which reduces vitamin K 2,3‐epoxide to active vitamin K.^32^, ^33^ Vitamin K is proved to be essential in bone development, including affecting the function of osteoblasts ^34^ and osteoclasts.^35^ The supplementation of vitamin K has an effect of osteoporosis prevention.^36^ Several studies also delineate that mutations of *VKORC1* are associated with bone mineral density and osteoporosis.^37^, ^38^ Thus, we hypothesized that hsa_circ_0006719 could contribute to the aetiology of CS by regulating the expression of *VKORC1*. Further functional studies are needed to identify this hypothesis.

There are several limitations in this study. First, since all the CS patients were under 18 years old, the age of the healthy controls was not matched with them both in the discovery cohort and in the replication cohort. The results could be biased by the unmatched age. Second, the circRNAs always act as post‐transcriptional regulators in target tissues. In our study, the circRNAs in plasma and tissues were not detected, and further studies are needed to explore the underlying mechanism. Third, the sample size of the study was relatively small. Further studies with larger sample size are necessary to validate our findings.

## 5 CONCLUSION

In conclusion, we described the first study of circRNAs in CS patients. After genome‐wide circRNAs analysis and validation in a replication cohort, we found that the differentially expressed circRNA‐related genes were enriched in several pathways, and we also found that hsa_circ_0006719 had a higher expression level in CS and could act as a potential novel diagnostic biomarker of CS.

## CONFLICT OF INTEREST

The authors declare no conflict of interest.

## AUTHOR CONTRIBUTION

GL and JS performed the research, analysed and interpreted the data. GL and JS drafted the manuscript. CC, YJ, ZL, HT, YL and TR helped sample collection. GL and CC performed helped analysis and interpretation of the data. HT provided technique support. JS offered professional discussions and instructions. CC, YL and TR helped bioinformatic analyses. JS conceived and designed the study, revised the manuscript and provided final approval of the manuscript.

## Supporting information

Table S1

Table S2

Table S3

Table S4

## Data Availability

Our data are available upon request to the corresponding author.

## References

[1] Sparrow DB , Chapman G , Smith AJ , et al. A mechanism for gene‐environment interaction in the etiology of congenital scoliosis. Cell. 2012;149(2):295‐306. 22484060 10.1016/j.cell.2012.02.054

[2] McMaster MJ , Ohtsuka K . The natural history of congenital scoliosis. A study of two hundred and fifty‐one patients. J Bone Joint Surg Am. 1982;64(8):1128‐1147. 7130226

[3] Shands AR Jr , Eisberg HB . The incidence of scoliosis in the state of Delaware; a study of 50,000 minifilms of the chest made during a survey for tuberculosis. J Bone Joint Surg. 1955;37(6):1243‐1249. 13271471

[4] Xue X , Shen J , Zhang J , et al. Rib deformities in congenital scoliosis. Spine. 2013;38(26):E1656‐E1661. 24343760 10.1097/BRS.0000000000000008

[5] Shen J , Zhang J , Feng F , Wang Y , Qiu G , Li Z . Corrective surgery for congenital scoliosis associated with split cord malformation: it may be safe to leave diastematomyelia untreated in patients with intact or stable neurological status. J Bone Joint Surg Am. 2016;98(11):926‐936. 27252437 10.2106/JBJS.15.00882

[6] Xue X , Shen J , Zhang J , et al. An analysis of thoracic cage deformities and pulmonary function tests in congenital scoliosis. Eur Spine J. 2015;24(7):1415‐1421. 24801576 10.1007/s00586-014-3327-6

[7] Al‐Kateb H , Khanna G , Filges I , et al. Scoliosis and vertebral anomalies: Additional abnormal phenotypes associated with chromosome 16p11.2 rearrangement. Am J Med Genet Part A. 2014;164(5):1118‐1126. 10.1002/ajmg.a.36401 24458548

[8] Wu N , Ming X , Xiao J , et al. TBX6 null variants and a common hypomorphic allele in congenital scoliosis. N Engl J Med. 2015;372(4):341‐350. 25564734 10.1056/NEJMoa1406829 PMC4326244

[9] Lin M , Zhao S , Liu G , et al. Identification of novel FBN1 variations implicated in congenital scoliosis. J Hum Genet. 2020;65(3):221‐230 31827250 10.1038/s10038-019-0698-x PMC6983459

[10] Li Z , Shen J , Wu WK , et al. Vitamin A deficiency induces congenital spinal deformities in rats. PLoS ONE. 2012;7(10):e46565. 23071590 10.1371/journal.pone.0046565 PMC3465343

[11] Hou D , Kang N , Yin P , Hai Y . Abnormalities associated with congenital scoliosis in high‐altitude geographic regions. Int Orthop. 2018;42(3):575‐581. 29387915 10.1007/s00264-018-3805-2

[12] Hansen TB , Wiklund ED , Bramsen JB , et al. miRNA‐dependent gene silencing involving Ago2‐mediated cleavage of a circular antisense RNA. EMBO J. 2011;30(21):4414‐4422. 21964070 10.1038/emboj.2011.359 PMC3230379

[13] Hansen TB , Jensen TI , Clausen BH , et al. Natural RNA circles function as efficient microRNA sponges. Nature. 2013;495(7441):384‐388. 23446346 10.1038/nature11993

[14] Memczak S , Jens M , Elefsinioti A , et al. Circular RNAs are a large class of animal RNAs with regulatory potency. Nature. 2013;495(7441):333‐338. 23446348 10.1038/nature11928

[15] Ashwal‐Fluss R , Meyer M , Pamudurti NR , et al. circRNA biogenesis competes with pre‐mRNA splicing. Mol Cell. 2014;56(1):55‐66. 25242144 10.1016/j.molcel.2014.08.019

[16] Legnini I , Di Timoteo G , Rossi F , et al. Circ‐ZNF609 is a circular RNA that can be translated and functions in myogenesis. Mol Cell. 2017;66(1):22‐37.e29. 28344082 10.1016/j.molcel.2017.02.017 PMC5387670

[17] Yang Y , Fan X , Mao M , et al. Extensive translation of circular RNAs driven by N(6)‐methyladenosine. Cell Res. 2017;27(5):626‐641. 28281539 10.1038/cr.2017.31 PMC5520850

[18] Li Y , Zheng Q , Bao C , et al. Circular RNA is enriched and stable in exosomes: a promising biomarker for cancer diagnosis. Cell Res. 2015;25(8):981‐984. 26138677 10.1038/cr.2015.82 PMC4528056

[19] Zhang SJ , Chen X , Li CP , et al. Identification and characterization of circular RNAs as a new class of putative biomarkers in diabetes retinopathy. Invest Ophthalmol Vis Sci. 2017;58(14):6500‐6509. 29288268 10.1167/iovs.17-22698

[20] Chen C , Tan H , Bi J , et al. Identification of competing endogenous RNA regulatory networks in vitamin A deficiency‐induced congenital scoliosis by transcriptome sequencing analysis. Cell Physiol Biochem: Int J Exp Cell Physiol Biochem Pharmacol. 2018;48(5):2134‐2146. 10.1159/000492556 30110682

[21] Zhou L , Chen J , Li Z , et al. Integrated profiling of microRNAs and mRNAs: microRNAs located on Xq27.3 associate with clear cell renal cell carcinoma. PLoS ONE. 2010;5(12):e15224. 21253009 10.1371/journal.pone.0015224 PMC3013074

[22] Love MI , Huber W , Anders S . Moderated estimation of fold change and dispersion for RNA‐seq data with DESeq2. Genome Biol. 2014;15(12):550. 25516281 10.1186/s13059-014-0550-8 PMC4302049

[23] Wilusz JE . A 360 degrees view of circular RNAs: from biogenesis to functions. Wiley Interdiscip Rev RNA. 2018;9(4):e1478. 29655315 10.1002/wrna.1478 PMC6002912

[24] Chen LL . The biogenesis and emerging roles of circular RNAs. Nat Rev Mol Cell Biol. 2016;17(4):205‐211. 26908011 10.1038/nrm.2015.32

[25] Yao Z , Luo J , Hu K , et al. ZKSCAN1 gene and its related circular RNA (circZKSCAN1) both inhibit hepatocellular carcinoma cell growth, migration, and invasion but through different signaling pathways. Mol Oncol. 2017;11(4):422‐437. 28211215 10.1002/1878-0261.12045 PMC5527481

[26] Chen CM , Lu YL , Sio CP , Wu GC , Tzou WS , Pai TW . Gene Ontology based housekeeping gene selection for RNA‐seq normalization. Methods (San Diego, CA). 2014;67(3):354‐363. 10.1016/j.ymeth.2014.01.019 24561167

[27] Kanehisa M , Araki M , Goto S , et al. KEGG for linking genomes to life and the environment. Nucleic Acids Res. 2007;36(Database):D480‐D484. 18077471 10.1093/nar/gkm882 PMC2238879

[28] Tian WX , Li JK , Qin P , et al. Screening of differentially expressed genes in the growth plate of broiler chickens with Tibial Dyschondroplasia by microarray analysis. BMC Genom. 2013;14:276. 10.1186/1471-2164-14-276 PMC3648502 23617778

[29] Hui S , Yang Y , Li J , et al. Differential miRNAs profile and bioinformatics analyses in bone marrow mesenchymal stem cells from adolescent idiopathic scoliosis patients. Spine J. 2019;19(9):1584‐1596. 31100472 10.1016/j.spinee.2019.05.003

[30] Klebig ML , Wall MD , Potter MD , Rowe EL , Carpenter DA , Rinchik EM . Mutations in the clathrin‐assembly gene Picalm are responsible for the hematopoietic and iron metabolism abnormalities in fit1 mice. Proc Natl Acad Sci USA. 2003;100(14):8360‐8365. 12832620 10.1073/pnas.1432634100 PMC166234

[31] Syx D , Malfait F , Van Laer L , et al. The RIN2 syndrome: a new autosomal recessive connective tissue disorder caused by deficiency of Ras and Rab interactor 2 (RIN2). Hum Genet. 2010;128(1):79‐88. 20424861 10.1007/s00439-010-0829-0

[32] Rishavy MA , Usubalieva A , Hallgren KW , Berkner KL . Novel insight into the mechanism of the vitamin K oxidoreductase (VKOR): electron relay through Cys43 and Cys51 reduces VKOR to allow vitamin K reduction and facilitation of vitamin K‐dependent protein carboxylation. J Biol Chem. 2011;286(9):7267‐7278. 20978134 10.1074/jbc.M110.172213 PMC3044983

[33] Rost S , Fregin A , Ivaskevicius V , et al. Mutations in VKORC1 cause warfarin resistance and multiple coagulation factor deficiency type 2. Nature. 2004;427(6974):537‐541. 14765194 10.1038/nature02214

[34] Ichikawa T , Horie‐Inoue K , Ikeda K , Blumberg B , Inoue S . Steroid and xenobiotic receptor SXR mediates vitamin K2‐activated transcription of extracellular matrix‐related genes and collagen accumulation in osteoblastic cells. J Biol Chem. 2006;281(25):16927‐16934. 16606623 10.1074/jbc.M600896200

[35] Kameda T , Miyazawa K , Mori Y , et al. Vitamin K2 inhibits osteoclastic bone resorption by inducing osteoclast apoptosis. Biochem Biophys Res Comm. 1996;220(3):515‐519. 8607797 10.1006/bbrc.1996.0436

[36] Braam LA , Knapen MH , Geusens P , et al. Vitamin K1 supplementation retards bone loss in postmenopausal women between 50 and 60 years of age. Calcif Tissue Int. 2003;73(1):21‐26. 14506950 10.1007/s00223-002-2084-4

[37] Holzer G , Grasse AV , Zehetmayer S , Bencur P , Bieglmayer C , Mannhalter C . Vitamin K epoxide reductase (VKORC1) gene mutations in osteoporosis: a pilot study. Translat Res: J Lab Clin Med. 2010;156(1):37‐44. 10.1016/j.trsl.2010.05.005 20621035

[38] Fodor D , Bondor C , Albu A , Popp R , Pop IV , Poanta L . Relationship between VKORC1 single nucleotide polymorphism 1173C>T, bone mineral density & carotid intima‐media thickness. Ind J Med Res. 2013;137(4):734‐741. PMC3724254 23703341

